# Complementary roles of virtual and real trials in medicine: An interplay of virtuous spirals

**DOI:** 10.1093/pnasnexus/pgag310

**Published:** 2026-09-16

**Authors:** Ehsan Samei, Liesbeth Vancoillie, Michael Wilde, Alejandro F Frangi, Ehsan Abadi, Predrag R Bakic, Kristina Bliznakova, Hilde Bosmans, Ann-Katherine Carton, Dimitra G Darambara, Paul Kinahan, Mojtaba Lashgari, Rie Tanaka, Hongkai Wang, Arezoo Zakeri

**Affiliations:** Center for Virtual Imaging Trials, Radiology, Duke University, 2424 Erwin Road, Suite 302, Durham, NC 27701, USA; Center for Virtual Imaging Trials, Radiology, Duke University, 2424 Erwin Road, Suite 302, Durham, NC 27701, USA; Kent and Medway Medical School, Pears Building, Parkwood Road, Canterbury CT2 7FS, Kent, United Kingdom; Division of Informatics, Imaging & Data Sciences, University of Manchester, Rm G530, Manchester M13 9PL, Greater Manchester, United Kingdom; Center for Virtual Imaging Trials, Radiology, Duke University, 2424 Erwin Road, Suite 302, Durham, NC 27701, USA; Department of Translational Medicine, Radiology Diagnostics, Lund University, Malmö 205 02, Skane, Sweden; Department of Medical Equipment, Electronic and Information Technologies in Healthcare, Medical University of Varna, Varna 9002, Bulgaria; Medical Physics & Quality Assessment, Department of Imaging & Pathology, KU Leuven, Leuven 3000, Flemish Brabant, Belgium; GE Healthcare, Buc 78530, Yvelines, France; Radiotherapy and Imaging – Multimodality Molecular Imaging, Institute of Cancer Research, London SW3 6JB, Greater London, United Kingdom; Department of Radiology, University of Washington, Seattle, WA 98195, USA; Institute of Biomedical Engineering, Department of Engineering Science, University of Oxford, Oxford OX3 7DQ, Oxfordshire, United Kingdom; Department of Radiological Technology, School of Health Sciences, College of Medical, Pharmaceutical and Health Sciences, Kanazawa University, Kanazawa 920-0942, Ishikawa, Japan; School of Biomedical Engineering, Faculty of Medicine, Dalian University of Technology, Dalian 116024, Liaoning Province, China; Division of Informatics, Imaging & Data Sciences, University of Manchester, Rm G530, Manchester M13 9PL, Greater Manchester, United Kingdom

**Keywords:** virtual imaging trials, in silico clinical trials, evidence pluralism (EBM+), digital twins in medicine

## Abstract

Virtual trials provide medical knowledge and practical methods for generating evidence through computational methods. But extending beyond those advantages, they offer epistemic opportunities to add mechanistic explainability to predictive inference of real trials. Relating the distinct contributions and ontology of virtual and real trials enables a transformative convergence of biological and clinical insights that they embody.

Significance statement This perspective highlights how virtual imaging trials and real clinical trials complement each other in generating medical evidence. It argues that simulations should be viewed not only as tools for efficiency but also as sources of mechanistic understanding that can inform and be informed by clinical data. By integrating virtual and real-world approaches within a single evidence ecosystem, this work supports a more flexible and context-driven framework for evaluating medical evidence and improving its translation into research, regulation, and clinical practice.

## Introduction

Virtual imaging trials (VITs), including in silico clinical trials and testing based on digital twins of patients and imaging systems, are transforming biomedical research, regulatory science, and patient care (1). The 2024 inaugural summit on Virtual Imaging Trials in Medicine (VITM24) laid the foundation for this transformation by articulating a shared vision of a guiding moonshot: a digital twin for every person, dynamically updated and ethically governed (2). The summit emphasized the practical imperatives of fostering robust simulations, standardizing validation frameworks, and multidisciplinary collaboration to translate this vision into reality (3).

Building upon these insights, this year's Virtual Imaging Trials in Medicine (VITM25) expanded the conversation to consider deeper epistemological and practical questions. These included how simulations contribute to knowledge, what counts as evidence beyond the clinical “gold standard,” how digital models should be evaluated, and whether simulations can play a justified evidential role. This framing positions virtual trials not merely as technical tools but as epistemic instruments that may reshape how evidence is defined and hierarchically organized in medicine. By exploring how clinical and virtual approaches complement each other and whether each is fit for its intended purpose, the discussion highlighted that both forms of evidence are interconnected and drive one another's progress. This relationship was described as a virtuous spiral in which data lead to models, models generate knowledge, knowledge informs experiments, and experiments produce additional data in an ongoing process across both real and virtual settings. This article summarizes the outcomes of VITM25, focusing on the epistemological and methodological contributions that VITs can offer to medicine.

## From efficiency to epistemology: the expanding role of simulation

VITs offer a significant documented advantage in operational efficiency in reducing the cost, time, and ethical challenges associated with traditional trials (3). Even so, viewing simulation purely in terms of its practical benefits is short-changing its existential value. Simulations are not merely convenient stand-ins for real-world experimentation; they are epistemic instruments in their own right. They allow for the articulation, testing, and refinement of medical hypotheses suited to the complexity of human biology and medical interventions. By modeling causal mechanisms, simulating counterfactual scenarios, or probing otherwise inaccessible phenomena, simulations offer new pathways to knowledge unavailable through more traditional means. For example, in intravoxel incoherent motion MRI, simulation enables the separation of Brownian and directional water molecule movements, the—effects of which cannot be distinguished empirically. Similarly, in AI-based imaging systems, simulations can help identify when model predictions are driven by spurious correlations rather than disease-relevant features. This provision uniquely addresses a significant gap in the development of assessments for transparent AI systems in medicine.

Motivated by a broader movement in the philosophy of evidence-based medicine (EBM), simulations further provide a pathway from abstract theories or models toward quantitative endpoints with clinical relevance, which can be empirically corroborated. The traditional EBM approach toward evidence in many contexts, including regulatory submissions, often relies upon an inflexible hierarchy of evidence. According to this hierarchy, evaluators should focus on real, randomized, clinical trials as the gold standard, and downplay other potential sources of evidence, such as studies into the underlying mechanisms of health and disease (4). This hierarchy is motivated by the perception that mechanistic studies oversimplify reality into simple models that fail to adequately represent the complexity of human biology or medical interventions. However, randomized clinical trials themselves embody simplified models, relying on presumed representative samples of target populations (5). As such, clinical trials alone may not always provide a complete understanding of treatment effects, as limitations in study populations, endpoints, duration, and real-world applicability can affect the generalizability and interpretation of findings.

It has been argued that mechanistic studies, which virtual trials fall into, are needed to help address the limitations of real clinical trials (4, 6, 7). For example, mechanistic studies can help determine whether the sample of individuals in a real clinical trial may be biased by comparing whether the mechanisms at play in the sample and target populations are sufficiently similar. Mechanistic studies, likewise, may be aided if complemented with real clinical trials; real-world data can serve as a benchmark to ascertain the validity of mechanistic models. The real and virtual/mechanistic approaches are therefore complementary rather than competitive.

The complementarity of real clinical trials and mechanistic studies has instigated a new approach to seeking evidence through so-called the EBM+ (8). EBM+ recognized a plurality of evidence from both real clinical trials *and* mechanistic studies, where simulations can support, refine, or in some cases substitute for more “traditional” sources of evidence. For example, in AI analysis of medical images, they can explain what known attributes of a disease, a priori modeled, may lead to unreliable predictions. Simulations are especially valuable where clinical experimentation is impractical, too costly, or unethical, such as trials involving high risk to the subjects. In those scenarios, they do not merely complement existing sources of evidence; they can expand the very boundaries of what counts as evidence in medicine. This expansion does not weaken evidentiary standards but shifts emphasis toward context-specific validation, transparency, and uncertainty characterization.

## Adequacy-for-purpose

As we consider virtual or real trials in terms of their abstract fidelity to reality, we should equally consider their adequacy for specific purposes. This view, articulated by Parker (9), reframes validation as contextual: a model is credible if it reliably serves its intended task. Task-centrality, user-centered design, and iterative feedback are therefore essential to epistemic robustness. In practice, adequacy is assessed through validity with respect to intended use, explicit uncertainty quantification, and relevance to the specific clinical or regulatory decision context. This shift also reflects the broader move away from traditional hierarchies of evidence and toward a more pluralistic approach to scientific inquiry embodied in the EBM+ approach.

The adequacy-for-purpose perspective has practical consequences for VITs. For regulatory submissions, for instance, developed simulations would need to meet different criteria than those used for hypothesis generation or education. A model supporting AI development may require robustness across clinical variability rather than maximal physical realism. Models need not be perfect to be valuable. Even simplified or seemingly flawed models, which may be dismissed as epistemically suspect, may still generate significant insights. As an example, MRI heating studies may offer insightful delineation of heating factors with a limited, imperfect model of the tissue without representing the cellular structure. Model limitations can thus function as productive drivers of scientific progress.

This flexible, purpose-oriented approach to evaluation aligns naturally with the recognition of simulation as not just a technical convenience, but as a dynamic contributor to knowledge-making. It underscores the importance of embedding simulation within an evidence ecosystem that values complementarity over uniformity, where robustness is achieved not through universal and inflexible standards, but through alignment of a model's purpose, structure, and context of use.

## Evaluation and clinical applicability

Simulations operate at the intersection of mechanistic understanding and empirical data. Their evaluation should therefore address both technical validity and practical utility, ensuring that virtual evidence meets expectations for safety, transparency, reproducibility, and clinical relevance across applications. Across applications ranging from AI development to regulatory support, the goal is not to replace clinical evidence but to complement and extend it.

Because medical VITs are designed to address clinically relevant questions, clinical applicability should be considered through their development rather than assessed only after completion. This requires close alignment between simulation objectives, modeling assumptions, and the realities of clinical decision-making and patient care. Clinicians already routinely rely on computational tools in diagnosis, treatment planning, and risk assessment; VIT therefore represents an extension of this broader movement toward computationally informed medicine.

Nevertheless, effective integration of VIT into practice depends on interdisciplinary collaboration. Simulation experts, clinicians, and biomedical researchers often approach evidence generation from a different methodological perspective and may prioritize different performance measures or endpoints. Early collaboration through joint research activities, codevelopment of study objectives, and iterative evaluation can help ensure that simulations address clinically meaningful needs and produce outcomes that are interpretable and actionable within healthcare settings.

Equally important is the involvement of patients as ultimate beneficiaries of biomedical innovation. Although one motivation of VIT is to reduce reliance on direct patient enrollment and alleviate associated ethical and logistical burdens, patient perspectives remain valuable. Beyond just participating as trial subjects, patients can help define clinically meaningful questions, identify relevant outcomes, inform implementation priorities, support translation into practice, and, overall, ensure our science is properly positioned to serve society. Incorporating patient voices in this way strengthens alignment between technological innovation, clinical priorities, and societal expectations.

## VIT and standardization

Data and model standardization are essential for democratized access to in silico resources. While data standardization traditionally involves data format and access, it must evolve to include workflow and contextual interpretability. The key question is not data availability, but fitness for the intended modeling purpose. This requires a structured framework that distinguishes between different processing tasks and informational layers, including raw measurement data, metadata, annotations, and clinical context. This also requires standardized and universally accessible, advanced modular pipelines within which different components from various developers can be integrated and utilized interoperably to conduct a trial using best-in-class data and processes.

The standardization should encompass definitions that span data and processes across the spectrum of the population, from cohorts to individuals. A layered typology of digital twins, ranging from generic population-based models to differential disease-specific models and fully personalized representations, provides a pathway toward scalable implementation while accommodating varying levels of data availability and purpose-specificity.

## Complementarity and the ecosystem of evidence

In the spirit of putting all facets of biomedical science toward advancing medicine, VITs are not intended to displace traditional sources of evidence (even though they might in certain situations), but to complement them within an integrated evidential ecosystem. In this evolving ecosystem, there exist real-world clinical patient data, synthetic data representing a population, and digital twin or virtual data representing individuals, each of which plays distinct yet interconnected roles. Rather than viewing them as competing, the emphasis should be on their complementarity, leveraging unique affordances to address different epistemic, clinical, and regulatory challenges. Each modality carries its own strengths and limitations. Real-world clinical patient data offer empirical grounding but remain subject to systematic biases, restricted generalizability due to nonrepresentative patient populations, and confounded factors outside the control of investigators. Synthetic data can fill specific gaps but may lack the full complexity or variability of actual individual patient cases—there is no such thing as an “average” person. Digital twins, derived from patient data, offer individual representation, control, scalability, and the ability to model counterfactuals, but can be simplistic and risk detachment from empirical reality if not adequately validated.

When used in isolation, each form of evidence may be insufficient. But when purposefully integrated, these approaches compensate for each other's limitations and biases, providing a more robust evidentiary foundation consistent with the EBM+ approach. Simulations, in particular, have the unique capacity to contextualize and interrogate real-world findings, to simulate longitudinal outcomes in rare or underrepresented populations, and to explore scenarios that are difficult or impossible to study empirically, such as multivariate treatment interactions. Regulatory sandboxing of simulations using digital twins can serve as scaffolds that make other forms of data more interpretable and actionable.

This integrated approach diverges from the traditional concept of a strict hierarchy of evidence, where consistent evidence (as demonstrated through systematic reviews of multiple randomized controlled clinical trials) is regarded as the most reliable form of evidence. Instead, aligned with EBM+, it embodies a flexible ecosystem where value is not determined by methodological purity alone, but by how well different types of evidence are transparently assessed for validity, uncertainty, and relevance to the decision context, and integrated to inform clinical reasoning and regulatory decision-making. The goal is not to rank types of evidence, but to use them *together* in a thoughtful and careful way toward clinical reasoning, regulatory decision-making, and public trust. By combining them effectively, we can create a stronger, more ethical, and more flexible foundation for modern medicine.

## The virtuous spirals of progress: rethinking knowledge in simulation

The epistemic structure of simulation-based knowledge challenges simple, linear models of scientific progress. Leonelli's model (10) describes scientific knowledge creation as a circle, a continuous loop of theory, modeling, data, and validation. Considering the progressive nature of knowledge, we submit that this process is better depicted as a spiral. In this model, each rotation cycles through the elements while building upon previous insights, progressively advancing our understanding over time. Crucially, interaction between the real and virtual spirals occurs through iterative feedback loops: empirical observations reveal model limitations, which trigger model refinement; simulations generate hypotheses and counterfactual scenarios that inform the design of new experiments; and discrepancies between simulated and observed outcomes drive validation and recalibration across both domains. In that form, the virtual/simulation methodology offers a similar and complementary line of progress (Fig. 1).

**Figure 1:**
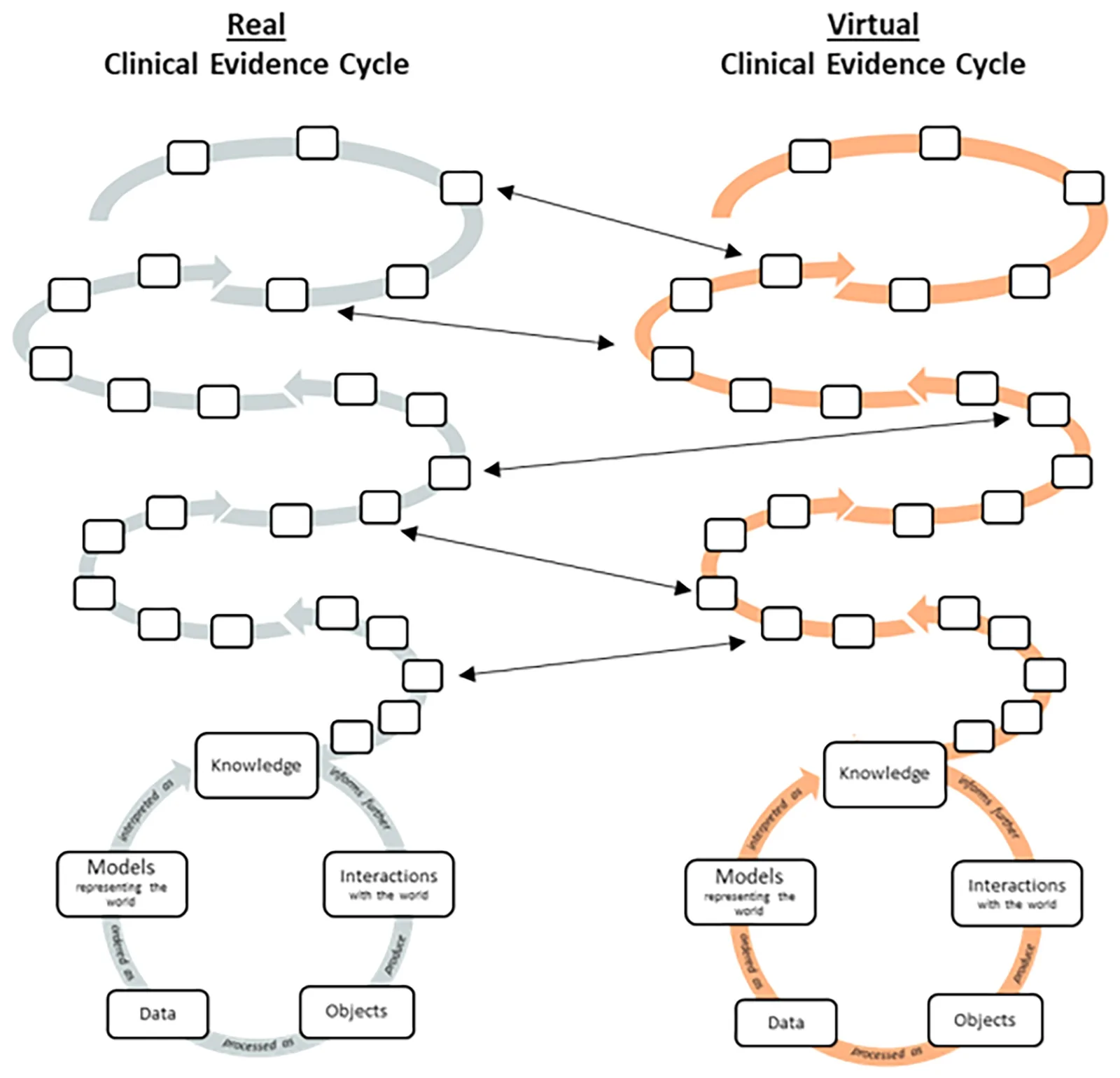
The circular and upward spiraling nature of real and virtual clinical evidence progress. This model illustrates the iterative and cumulative nature of knowledge creation through complementary approaches. Each cycle integrates theoretical development, computational modeling, data generation, and empirical validation. The circular cycle underlying the spiral representation is adapted from Leonelli's model of scientific knowledge creation. The spiral shape emphasizes progressive refinement and expanding insight over time, highlighting the nonlinear and dynamic nature of progress. The connection between the two virtuous spirals indicates how the two progressions and their components inform, enrich, and reinforce one another.

This spiral model captures the dynamic and ongoing nature of scientific progress and the role of simulation in that progress. Rather than simply applying existing knowledge, simulations refine, challenge, and generate new insights at every turn. Knowledge is not fixed but evolves through continuous interaction between theoretical assumptions, model structures, empirical measurements, and real-world contexts. Importantly, this ongoing process removes the idea that any single element, whether real or simulated, and whether theory, model, or data, holds ultimate authority. Instead, their credibility and relevance appear only through continuous interaction, validation, and refinement.

The virtuous spiral model of progress calls for a more humble and reflective scientific practice that recognizes models and real trials as provisional and embraces the ethical responsibilities involved in their use. Such humility is especially important in the case of digital twins of patients, which promise highly personalized medicine but also raise serious ethical and legal questions. Issues around data ownership, consent, interpretability, and trust must be addressed not as afterthoughts but as fundamental parts of the design, development, and governance of virtual trial systems. Only by including these considerations from the beginning can the potential of simulation-based medicine be realized responsibly and ethically.

## Scopes of applicability and stakeholders

VITs span imaging domains from breast imaging to cardiac and MRI, necessitating interdisciplinary collaboration among scientists (including biologists and biochemists), clinical partners (including clinicians in oncology, cardiology, neurology, and geriatrics), and healthcare manufacturers as integral contributors to the healthcare ecosystem, thereby grounding VIT in the best of science, technology, and routine care practices and decision-making contexts. As noted earlier, equally important is integrating perspectives from patients and patient advocacy groups, which can help align technical progress with societal priorities and patient needs. Supporting common infrastructures, such as federated digital twin repositories and privacy-preserving simulation platforms, is essential for responsible model sharing, reproducibility, and scalability.

VIT's applicability depends not only on computational prowess or regulatory alignment, but also on a commitment to evidential pluralism and a collective willingness to collaborate across disciplinary boundaries with genuine intellectual curiosity, acknowledging that each discipline, whether rooted in mathematical modeling, biological processes, clinical observation, regulatory science, or patient experience, offers radically different yet equally valid and vital perspectives on the complex phenomenon of human health. This interdisciplinary integration also motivates ethical reflection, as VIT influences how evidence is generated, validated, and ultimately used in clinical and regulatory decision-making, with implications for transparency, accountability, and bias. The computational scientist's algorithmic precision, the clinician's embodied knowledge, the ethicist's moral reasoning, and the patient's lived experience each illuminate aspects of reality that might be invisible to the others. True progress emerges not from the dominance of any single worldview, but from the patient work of weaving these diverse epistemic contributions into a richer, more nuanced understanding that honors the irreducible complexity of human biology and the healthcare systems that serve it. Only through such a humble interdisciplinary approach can we hope to realize virtual trials that are not merely technically sophisticated but genuinely transformative in their capacity to improve human health. The foundational questions and key insights discussed throughout this perspective are summarized in Table 1.

**Table 1:** Foundational questions and answers on the role of VITs in medicine.

Short question	Short answer
1. How do simulations and in silico models and data contribute to medical knowledge?	They act as epistemic instruments that refine, challenge, and generate explainable insights beyond what real trials alone can provide in terms of inference and real-world evidence.
2. What counts as evidence in medicine when randomized control trials are treated as the gold standard?	Evidence is pluralistic: simulations, mechanistic studies, and real trials are complementary as they enrich and inform one another in virtuous spirals of progress toward a stronger evidential base.
3. How should we evaluate digital models?	By their validation against empirical adequacy-for-purpose in addition to fidelity-to-reality; models are credible when they reliably serve their intended task.
4. Can simulations play an evidential role with philosophical justification?	Yes, simulations expand what counts as evidence as aligned with the EBM+ movement in philosophy of medicine.
5. What is the role of standardization of virtual trials?	They ensure interoperability, usability, and scalability across data, models, and workflows.
6. How can simulations be translated into clinical practice?	Through shared ontological frameworks, vernaculars, interdisciplinary collaboration, and inclusion of patient voices.
7. What ethical issues must be addressed?	Ownership, consent, interpretability, and trust must be built into design and governance from the start.
8. How should we understand scientific progress in the era of simulation?	As a virtuous spiral: iterative, complementary, and humble, rather than linear or hierarchical.

The integration of VIT into medical decision-making also raises governance considerations. Simulation developers, clinicians, regulators, and patients operate under different incentives and levels of interpretability, and these asymmetries may influence how evidence is produced and used. In particular, highly technical models may not be independently interpretable by all stakeholders, underscoring the need for a governance structure that includes transparent validation frameworks and clear allocation of responsibility in decision-making.

## Conclusions

VITs constitute a unique methodology to obtain medical knowledge, not only through enhanced efficiency and practicality enabled by computational methods but also by their ability to interrogate the underlying biological and interventional processes mechanistically; VITs are not merely instruments of efficiency—they are sites of epistemic innovation. Their advancement requires epistemological clarity alongside technical and regulatory developments. Their distinct advantage should not be measured only in terms of impeccable fidelity-to-reality, which is not attainable perfectly by any scientific means, but also adequacy-for-purpose within complementary evidence systems where real and virtual trials can enrich and inform one another. This prospect is possible by not just technical sophistication, but by an intellectual humility that recognizes each discipline's irreducible contributions to understanding the complexity of human health, and by a commitment to relate disciplinary vernaculars and ontologies that are rooted in distinct epistemic paradigms. This can provide a framework through which biological realism, analytical insights, clinical wisdom, regulatory rigor, patient experience, and interdisciplinary cooperation can meaningfully converge in the transformative advancement of human health.

## Data Availability

This manuscript is a perspective article and does not report new data generation or analysis. No datasets were created or analyzed in this study. All concepts and arguments are based on previously published literature, which is cited in the reference list. No code was used for data analysis.
